# Prognostic impact of organ involvement in aggressive adult T-cell leukemia/lymphoma: definition of risk organ and proposal of a prognostic index

**DOI:** 10.1038/s41408-025-01367-w

**Published:** 2025-10-16

**Authors:** Koji Jimbo, Ayumu Ito, Hirona Ichimura, Junichi Kuroda, Shohei Andoh, Aki Sato, Kazuaki Yokoyama, Takahiro Fukuda, Kaoru Uchimaru, Yasuhito Nannya

**Affiliations:** 1 Division of Hematopoietic Disease Control, The Institute of Medical Science, The University of Tokyo, Tokyo, Japan; 2 Department of Hematology/Oncology, Research Hospital, The Institute of Medical Science, The University of Tokyo, Tokyo, Japan; 3 Department of Hematopoietic Stem Cell Transplantation, National Cancer Center Hospital, Tokyo, Japan

**Keywords:** T-cell lymphoma, Tumour virus infections, Chemotherapy, Cancer therapeutic resistance

**To the Editor:**

Adult T-cell leukemia/lymphoma (ATL) is a lymphoma with a very poor prognosis, originating from the tumorigenesis of human T-cell leukemia virus (HTLV)-1-infected T lymphocytes [1, 2]. The acute, lymphoma, and chronic types with unfavorable factors are categorized as aggressive ATL, which requires intensive systemic therapy including allogeneic hematopoietic cell transplantation (allo-HCT), and has a particularly poor prognosis [3, 4]. Several disease-specific prognostic models have been proposed for aggressive ATL: prognostic index for ATL (ATL-PI), modified ATL-PI, and Japan Clinical Oncology Group prognostic index (JCOG-PI) [5–7]. Among them, the widely used simplified ATL-PI emphasizes clinical stage [5], and it is recognized that advanced clinical stage is associated with poor prognosis within aggressive ATL. However, in several large study cohorts used to construct these prognostic indices, more than 90% of aggressive ATL cases were reported to be in advanced clinical stage (≥stage III), with many in stage IV [5–7]. Given the high number of stage IV cases, further subdivision is important for more detailed prognostic stratification of aggressive ATL.

It is also recognized that extranodal involvement is more prevalent in ATL than in other lymphomas [5–12]. However, the details of organ involvement profile have not been fully clarified. Furthermore, aggressive ATL cases with central nervous system (CNS) involvement, as well as those with a high burden of ATL cells in peripheral blood, have been reported to have poor prognosis [13, 14], but the association between other specific organ involvement and prognosis has not been reported. Considering this background, we explored whether organ involvement profiles could further stratify stage IV cases into subgroups with distinct prognoses.

This single-center, retrospective study included 140 patients diagnosed with aggressive ATL and treated with systemic therapy. The characteristics of the cases are summarized in Table S1, and details of the research methods are summarized in supplemental materials. The median age at the start of treatment was 62 years (range, 28–82), and 49% were male. Among patients with available data for each relevant item, 81% had an Eastern Cooperative Oncology Group performance status (ECOG PS) of 0–1, 99% were clinical stage IV, and 85% had acute-type ATL. Sixty-five percent of patients received vincristine, cyclophosphamide, doxorubicin, and prednisolone (VCAP)–doxorubicin, ranimustine, and prednisolone (AMP)–vindesine, etoposide, carboplatin, and prednisolone (VECP) as initial systemic therapy, and 59% received allo-HCT during the course of treatment. The median overall survival (OS) was 20.8 months (95% confidence interval [CI], 14.7–31.4 months), and the 2-year OS was 48.2% (95% CI, 39.3–56.5%) in the entire cohort.

We investigated the status of organ involvement in aggressive ATL. Detailed definitions of each organ involvement are shown in Table S2 but briefly described as follows. Peripheral blood lesions were defined as abnormal lymphocytes ≥5%. Cases with pathological evidence of ATL cell infiltration in a specific organ were defined as positive for involvement of that organ. Cases with abnormal imaging findings or organ dysfunction compatible with ATL involvement that improved in response to ATL treatment were also considered involvement-positive for the affected organs. Excluding cases with unavailable data, the percentages of cases with positive organ involvement were as follows: peripheral blood 78%, bone marrow 67%, lymph nodes 90%, skin 56%, lung 27%, liver 50%, spleen 48%, CNS 20%, gastrointestinal tract 16%, pleural effusion 11%, ascites 8%, kidney 4%, pancreas 3%, muscle 2%, pleura 2%, and peritoneum 2% (Tables S1, S3). Among these ATL lesions, cases with positive involvement of the lung, liver, spleen, CNS, and pleura had significantly shorter OS compared with cases without involvement (Fig. 1A–D, Fig. S1). CNS and lung involvement were associated with poor ECOG PS, high-risk group of simplified ATL-PI, ATL-associated death, and a lower likelihood of receiving allo-HCT (Fig. S2A–C, Table S3). Even among patients ≤70 years of age, generally considered eligible for allo-HCT, fewer patients with CNS and lung lesions received allo-HCT compared to patients with other organ involvement (Fig. S2D, Table S3). Poor ECOG PS may explain the low proportion of patients receiving allo-HCT. When patients were divided into three groups (≤3, 4–5, and ≥6 lesions among 16 organs), OS was significantly worse in the group with more organ lesions (*P* < 0.001) (Fig. 1E). The ≤3, 4–5, and ≥6 lesions groups had median OS of not reached (NR) (95% CI, 46.8 months–NR), 34.8 months (95% CI, 13.3–NR), and 10.0 months (95% CI, 8.2–14.7), respectively, and 2-year OS of 81.9% (95% CI, 58.6–92.8), 67.8% (95% CI, 48.3–81.3), and 10.1% (95% CI, 1.8–27.0), respectively. These results suggest that the number of involved organs negatively impacts OS in aggressive ATL, with varying effects depending on the involved organ.

**Fig. 1:**
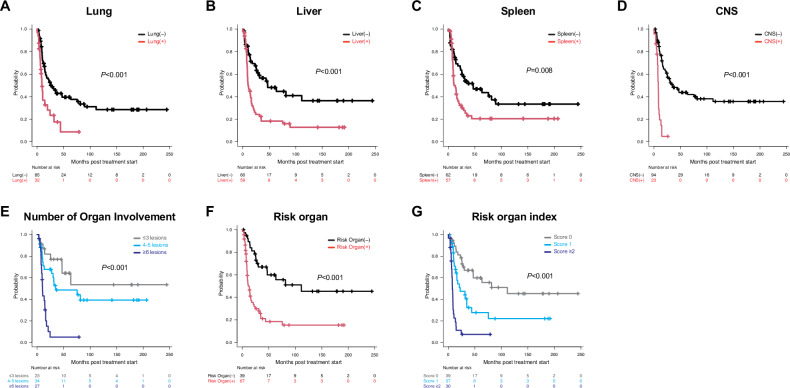
Overall survivals of each organ involvement profile. Overall survival of positive and negative groups for each organ lesion: (**A**) Lung; (**B**) Liver; (**C**) Spleen; (**D**) Central nervous system (CNS). **E** Overall survival of three groups by number of involved organs: ≤3, 4–5, and ≥6 lesions. **F** Overall survival of positive and negative groups for risk organ involvement (lung, liver, and CNS). **G** Overall survival of three groups by risk organ index score (0, 1, and ≥2): 2 points assigned for CNS involvement and 1 point each for lung and liver involvement. All *P* values were calculated using the log-rank test.

We then investigated the detailed effects of organ involvement on OS. Of the five significant organ lesions in the univariate analysis (lung, liver, spleen, CNS, and pleura), pleura was excluded due to low incidence, and the remaining four organs were subjected to multivariate analysis of OS. Three organ lesions—excluding the spleen—remained significant factors for OS (Table S4). Therefore, we defined the lung, liver, and CNS as risk organs that predict poor OS in aggressive ATL. The presence of risk organ involvement significantly stratified the cohort in terms of OS (*P* < 0.001; hazard ratio 3.35, 95% CI 1.90–5.89) (Fig. 1F). Additionally, cases with splenic involvement had a higher percentage of involvement of the three risk organs (Fig. S3). This is one reason why the spleen was not a risk organ. Subsequently, a multivariate analysis was performed to further evaluate the effect of risk organs on OS in the presence of known significant factors (age, ECOG PS, ATL subtype, serum albumin level, soluble interleukin-2 receptor level, corrected serum calcium level, and C-reactive protein level). These additional factors are components of existing prognostic indexes (simplified ATL-PI, modified ATL-PI, and JCOG-PI) [5–7]. As a result, the presence or absence of risk organs remained the only factor influencing OS (Table S5). These results indicate that risk organ involvement is a significant prognostic factor in aggressive ATL.

Finally, we constructed a new prognostic model for aggressive ATL focusing on risk organ involvement. Based on the hazard ratios from the multivariate analysis (Table S4), the total score for each case was calculated with one point each for lung and liver involvement and two points for CNS involvement. This classification divided the 106 cases with available data on risk organ involvement into three groups: 39 cases in the score 0 group, 37 cases in the score 1 group, and 30 cases in the score ≥ 2 group. These groups had a median OS of 111.4 months (95% CI, 30.1–NR), 22.2 months (95% CI, 13.4–34.9), and 7.6 months (95% CI, 6.1–8.9), respectively, and 2-year OS of 78.5% (95% CI, 61.6–88.6), 47.5% (95% CI, 29.5–63.5), and 11.3% (95% CI, 2.9–26.3), respectively. Thus, these three groups significantly stratified the cohort in terms of OS (*P* < 0.001) (Fig. 1G). To evaluate the usefulness of the prognostic index developed in this study (i.e., the risk organ index for aggressive ATL), we compared it with existing prognostic indices (simplified ATL-PI, modified ATL-PI, and JCOG-PI) [5–7]. In this cohort, simplified ATL-PI (*P* < 0.001) and JCOG-PI (*P* = 0.013) significantly stratified prognosis, while modified ATL-PI was not a significant index (*P* = 0.085) (Fig. S4). Comparing the concordance indices (C-indices) of these prognostic models, the risk organ index for aggressive ATL constructed in this study had the highest C-index (Table 1). However, there are concerns regarding the uniform comparison of these prognostic factors. Previous prognostic models were established for restricted cohorts: the simplified ATL-PI cohort excluded allo-HCT cases; the modified ATL-PI and JCOG-PI cohorts were limited to patients aged ≤70 years and <70 years, respectively, assuming allo-HCT eligibility [5–7]. This difference in cohort composition may explain the insufficient prognostic separation seen with existing prognostic models in this cohort. Therefore, the C-index was recalculated under the conditions in which each model was originally developed for a proper comparison of prognostic efficacy. Even so, this novel prognostic model outperformed the C-index of each existing model under all limited conditions for which the existing prognostic models were established (Table 1). These results suggest that risk organ involvement profiles in aggressive ATL may serve as a promising prognostic model.

**Table 1:** Concordance index of each prognostic model for aggressive ATL.

Model	Cohort	C-index	95%CI
Risk organ index for aggressive ATL	All	0.72	0.69–0.75
	cases without allo-HCT	0.72	0.68–0.77
	cases with ≤70 years	0.70	0.67–0.73
	cases with <70 years	0.71	0.67–0.74
Simplified ATL-PI	All	0.64	0.61–0.67
	cases without allo-HCT	0.55	0.50–0.59
Modified ATL-PI	All	0.55	0.53–0.58
	cases with ≤70 years	0.54	0.52–0.57
JCOG-PI	All	0.56	0.54–0.59
	cases with <70 years	0.56	0.53–0.59

*allo-HCT* allogeneic hematopoietic cell transplantation, *ATL* adult T-cell leukemia/lymphoma, *JCOG* Japan Clinical Oncology Group, *PI* prognostic index.

This study had several limitations. First, it was a single-center, retrospective study. A multicenter, prospective study is desirable. Second, some data on organ lesions were missing, so prospectively collected data would be preferable. Third, there was no validation cohort. Although this was a large cohort for rare ATL cases, validation is necessary to confirm that the results are universally applicable. Finally, the novel prognostic model accounts only for risk organ involvement. Developing a more multilayered prognostic model that includes each risk organ separately would be ideal but will require larger cohorts.

In summary, we identified risk organs based on the detailed organ involvement profile of aggressive ATL and developed a novel prognostic model in this study. Validation of this model in a larger cohort is warranted.

## Supplementary information

Supplemental figures

Supplemental tables

Supplemental methods and discussion

## Data Availability

The data that support the findings of this study are available from the corresponding author upon reasonable request.
