# Supplementary material for: Prognostic impact of organ involvement in aggressive adult T-cell leukemia/lymphoma: definition of risk organ and proposal of a prognostic index
Source: Blood Cancer J. 2025 Oct 16;15(1):166. doi: 10.1038/s41408-025-01367-w (PMC12533117; doi:10.1038/s41408-025-01367-w)
Supplement: Supplementary file 1 — Supplemental figures [file 41408_2025_1367_MOESM1_ESM.pdf]

Figure S1

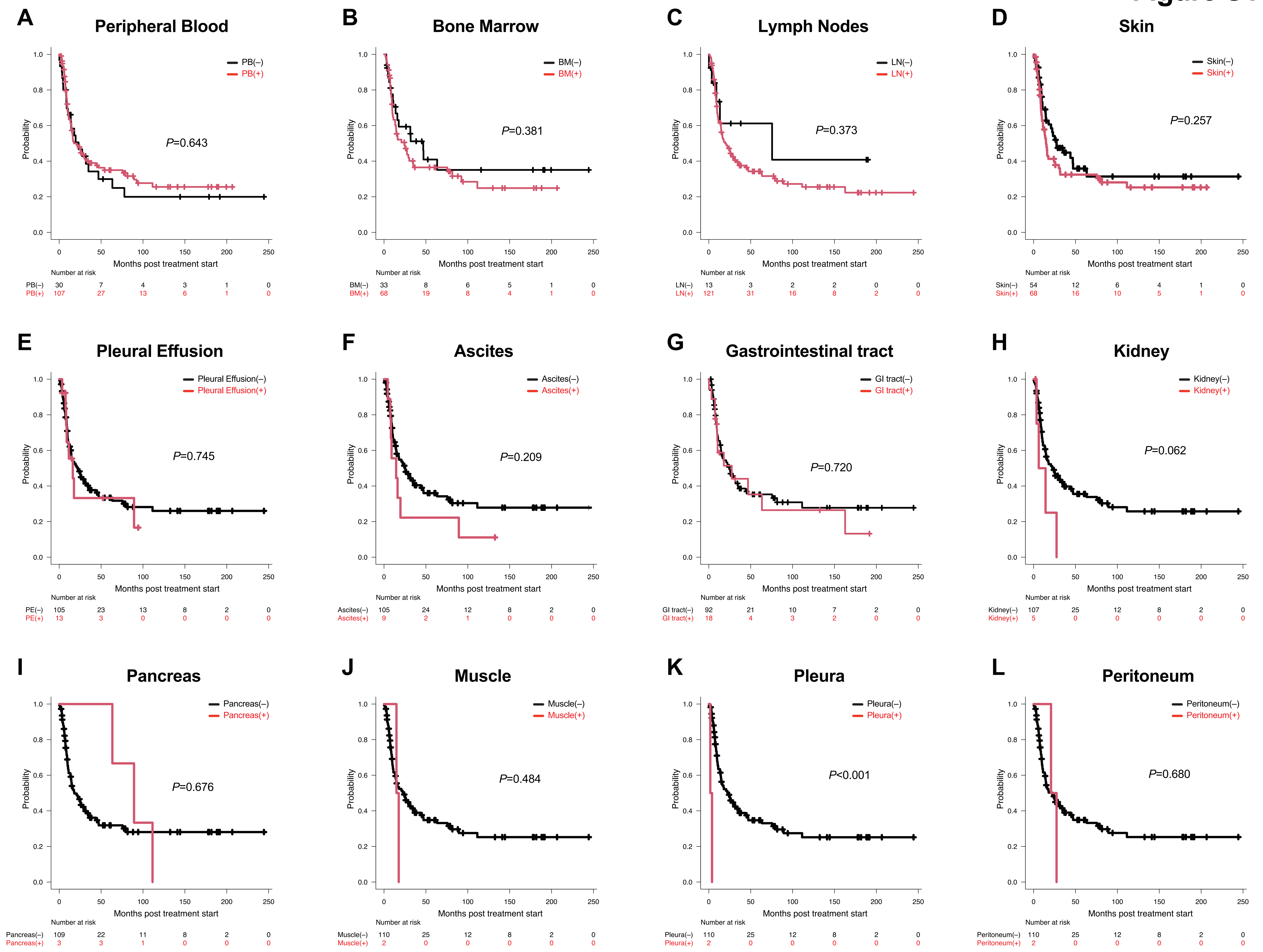

Supplementary Figure 1. Prognosis of specific organ involvement profiles
(A-L) Overall survival of positive and negative groups for each organ lesion; (A) Peripheral blood; (B) Bone marrow; (C) Lymph nodes; (D) Skin; (E) Pleural effusion; (F) Ascites; (G) Gastrointestinal tract; (H) Kidney; (I) Pancreas; (J) Muscle; (K) Pleura; (L) Peritoneum.

Figure S2

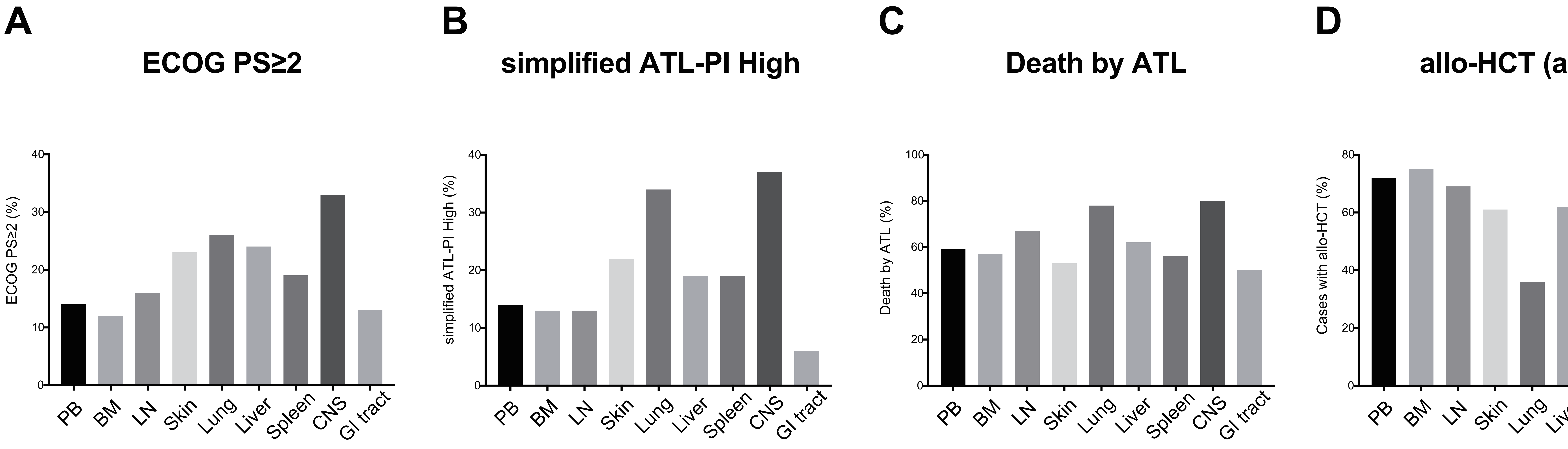

Supplementary Figure 2. Percentage of applicable cases in each organ lesion–positive group:
(A) Eastern Cooperative Oncology Group performance status (ECOG PS) ≥ 2; (B) High risk according to the simplified ATL-PI;
(C) ATL-associated deaths; (D) Allogeneic hematopoietic cell transplantation (allo-HCT) performed among patients ≤70 years of age.
PB, peripheral blood; BM, bone marrow; CNS, central nervous system; GI tract, gastrointestinal tract.

Figure S3

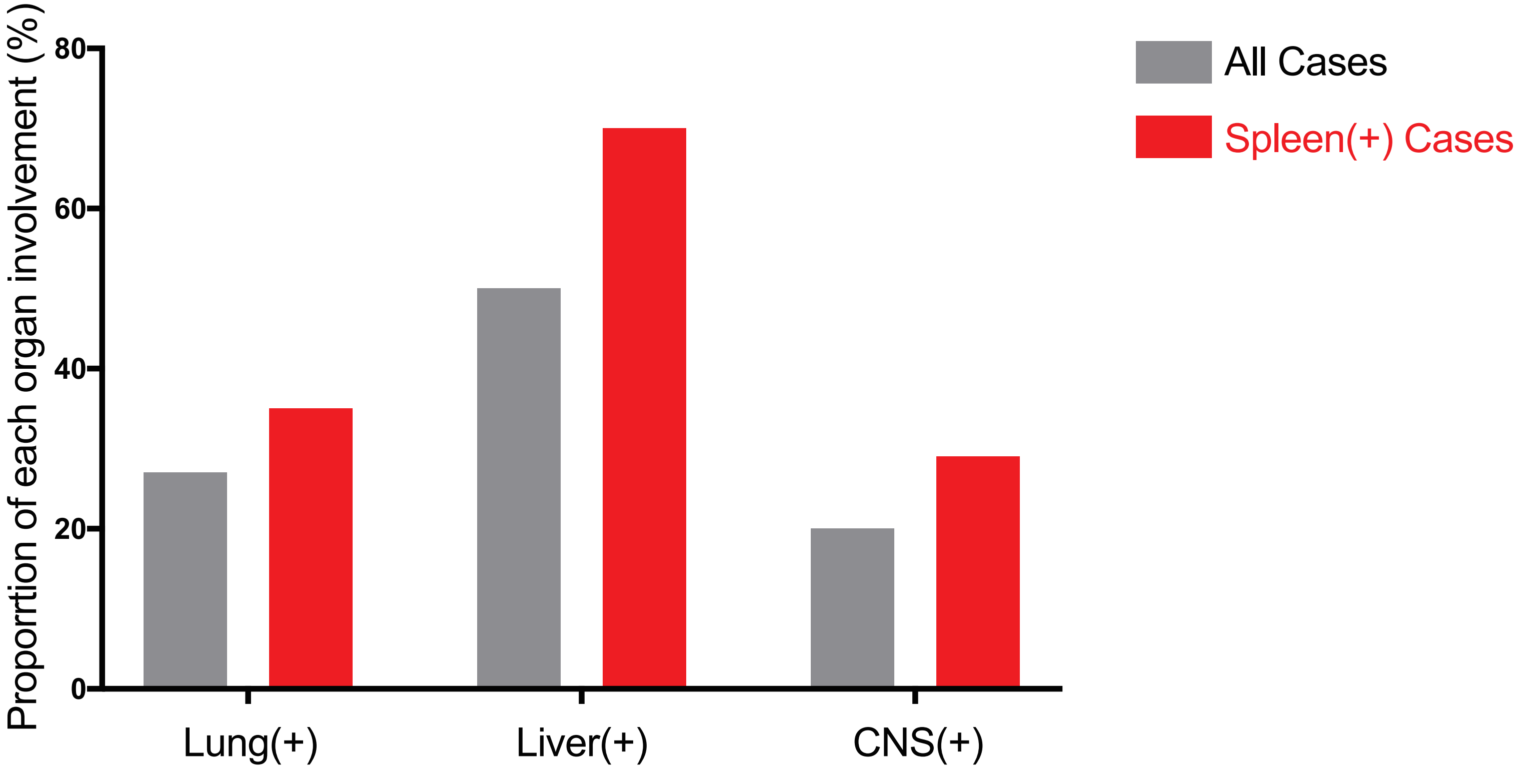

Supplementary Figure 3.
Percentage of each risk organ positive in cases with positive for splenic involvement compared to all cases.

Figure S4

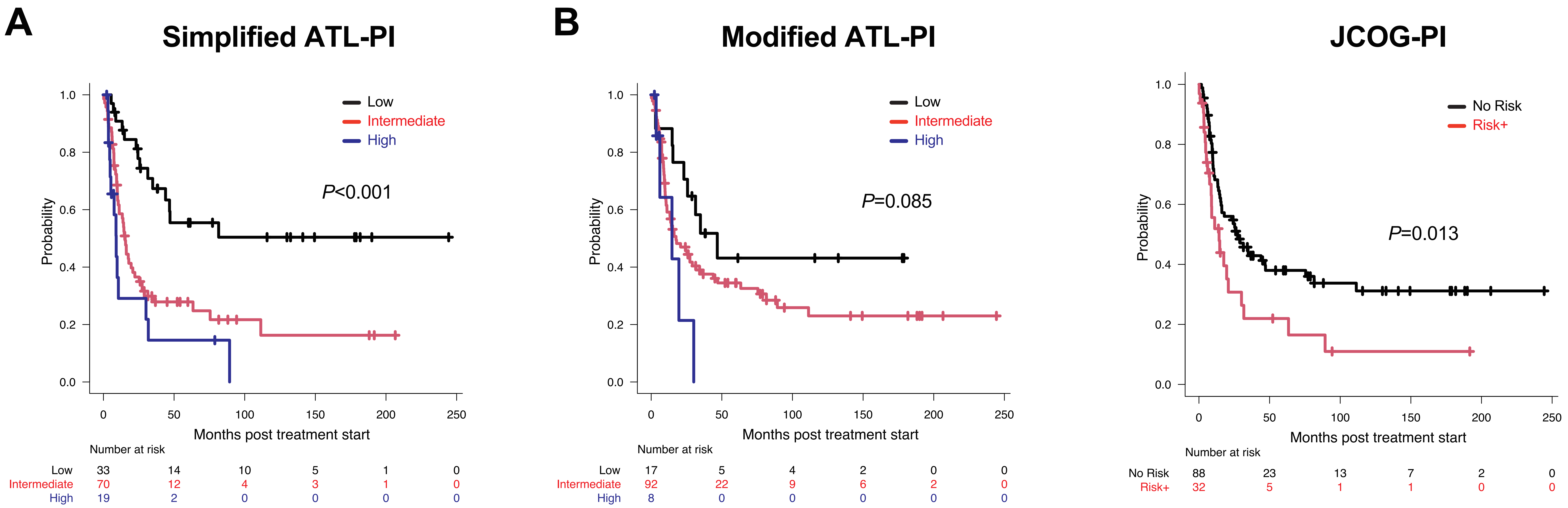

Supplementary Figure 4.
Overall survival in this cohort by existing prognostic models specific to ATL; (A) Simplified ATL-PI; (B) Modified ATL-PI; (C) JCOG-PI.
