# Supplementary material for: Prognostic impact of organ involvement in aggressive adult T-cell leukemia/lymphoma: definition of risk organ and proposal of a prognostic index
Source: Blood Cancer J. 2025 Oct 16;15(1):166. doi: 10.1038/s41408-025-01367-w (PMC12533117; doi:10.1038/s41408-025-01367-w)
Supplement: Supplementary file 2 — Supplemental tables [file 41408_2025_1367_MOESM2_ESM.docx]

Table S1. Characteristics of all patients with aggressive ATL in this study (n=140).

| Characteristics | Value | Evaluable Number | % | range |
| --- | --- | --- | --- | --- |
| Median age at the start of initial treatment, years | 62 | 140 |  | 28-82 |
| Sex, male | 68 | 140 | 49 |  |
| ECOG PS |  | 124 |  |  |
| 0-1 | 100 |  | 81 |  |
| ≥2 | 24 |  | 19 |  |
| Stage IV | 137 | 139 | 99 |  |
| Simplified ATL-PI |  | 122 |  |  |
| Low | 33 |  | 27 |  |
| Int | 70 |  | 57 |  |
| High | 19 |  | 16 |  |
| ATL subtype at the start of initial treatment |  | 140 |  |  |
| Chronic (Unfavorable) | 8 |  | 6 |  |
| Lymphoma | 13 |  | 9 |  |
| Acute | 119 |  | 85 |  |
| Organ involvement |  |  |  |  |
| Peripheral blood | 107 | 137 | 78 |  |
| Bone marrow | 68 | 101 | 67 |  |
| Lymph nodes | 121 | 134 | 90 |  |
| Skin | 68 | 122 | 56 |  |
| Lung | 32 | 117 | 27 |  |
| Liver | 59 | 119 | 50 |  |
| Spleen | 57 | 119 | 48 |  |
| Central nervous system | 23 | 117 | 20 |  |
| Gastrointestinal tract | 18 | 110 | 16 |  |
| Pleural effusion | 13 | 118 | 11 |  |
| Ascites | 9 | 114 | 8 |  |
| Kidney | 5 | 112 | 4 |  |
| Pancreas | 3 | 112 | 3 |  |
| Muscle | 2 | 112 | 2 |  |
| Pleura | 2 | 112 | 2 |  |
| Peritoneum | 2 | 112 | 2 |  |
| Number of organ involvement |  | 84 |  |  |
| ≤3 | 23 |  | 27 |  |
| 4-5 | 34 |  | 40 |  |
| ≥6 | 27 |  | 32 |  |
| Initial systemic therapy for aggressive ATL |  | 140 |  |  |
| VCAP-AMP-VECP | 91 |  | 65 |  |
| CHOP like regimen | 22 |  | 16 |  |
| Mogamulizumab ± chemotherapy | 17 |  | 12 |  |
| Other | 10 |  | 7 |  |
| Median value of each data |  |  |  |  |
| sIL-2R, U/mL | 19,035 | 124 |  | 381-192,231 |
| LDH, IU/L | 508 | 129 |  | 119-4,213 |
| Serum albumin, g/dL | 3.4 | 124 |  | 1.8-4.7 |
| Corrected Ca level, mg/dl | 9.8 | 123 |  | 8.4-17.7 |
| C-reactive protein, mg/dL | 0.59 | 121 |  | 0.01-16.41 |
| Cases with allo-HCT | 82 | 140 | 59 |  |
| Median follow-up time, months | 15 | 140 |  | 0-244 |
| Median overall survival, months (95% confidence interval) | 20.8 | 140 |  | (14.7-31.4) |
| Two-year overall survival, % (95% confidence interval) | 48.2 | 140 |  | (39.3-56.5) |

allo-HCT, allogeneic hematopoietic cell transplantation;ATL, adult T-cell leukemia/lymphoma; Ca, calcium; CHOP, cyclophosphamide, vincristine, doxorubicin, and prednisolone; ECOG PS, Eastern Cooperative Oncology Group performance status; LDH, lactate dehydrogenase; PI, prognostic index; sIL-2R, soluble interleukin-2 receptor; VCAP, vincristine, cyclophosphamide, doxorubicin, and prednisolone, -AMP, doxorubicin, ranimustine, and prednisolone, -VECP, vindesine, etoposide, carboplatin, and prednisolone.

Table S2. Definitions of each organ involvement.

| Organ | Definitions |
| --- | --- |
| Peripheral Blood | Cases with ≥5% of abnormal lymphocytes in peripheral blood. |
| Bone Marrow | Cases with positive bone marrow infiltration of ATL cells on bone marrow smear or pathological evaluation. |
| Lymph Nodes | Cases with pathological findings of T-cell lymphoma on lymph node biopsy. |
|  | Cases with imaging findings such as enlarged lymph nodes or FDG accumulation, and considered positive lymph node involvement based on clinical course including response to treatment. |
| Skin | Cases with pathological findings of ATL cell infiltration on skin biopsy |
|  | Cases with skin rash and considered positive skin involvement based on clinical course including response to treatment. |
| Lung | Cases with pathological findings of ATL cell infiltration in lung biopsy or in bronchoalveolar lavage. |
|  | Cases with imaging findings such as lung lesions or FDG accumulation, and those who are considered to have lung involvement based on the clinical course including response to treatment. |
| Liver | Cases with hepatomegaly (>18 cm in vertical diameter) on imaging studies (echography or CT) and considered positive liver involvement based on clinical course including response to treatment. |
|  | Cases with alanine aminotransferase >3.0 times of upper limit of normal or total bilirubin >1.5 times of upper limit of normal and considered positive liver involvement based on clinical course including response to treatment. |
| Spleen | Cases with splenomegaly (maximum diameter >12 cm) on imaging studies (echography or CT). |
|  | Cases with with splenomegaly ≤12 cm but considered to have positive spleen involvement based on clinical course including response to treatment |
| Central Nervous System | Cases with cytology Class IV* or higher on cerebrospinal fluid examination. |
|  | Cases with intracranial lesions on imaging studies (magnetic resonance imaging) and diagnosed as ATL central nervous system lesions. |
| Gastrointestinal tract | Cases with pathological findings of ATL cell infiltration on gastrointestinal tract biopsy. |
|  | Cases with imaging findings such as gastrointestinal tract lesions or FDG accumulation, and considered to have positive gastrointestinal tract involvement based on clinical course including response to treatment. |
| Pleural Effusion Ascites | Cases with cytology Class IV* or higher on each fluid examination |
|  | Cases with imaging findings such as fluid retention or FDG accumulation, and considered to have positive ATL cell involvement based on clinical course including response to treatment |
| Kidney Pancreas Muscle Preura Peritoneum | Cases with pathological findings of ATL cell infiltration on each organ biopsy. |
|  | Cases with imaging findings such as each organ lesions or FDG accumulation, and considered positive each organ involvement based on clinical course including response to treatment. |

*Pathological evaluation was performed in accordance with the standard Papanicolaou classification system as follows: class I, normal; class II, benign atypia; class III, suggestive of malignancy; class IV, strongly suggestive of malignancy; and class V, consistent with malignancy.

ATL, adult T-cell leukemia/lymphoma; CT, computed tomography; FDG, fluorodeoxyglucose

Table S3. Characteristics of patients with aggressive ATL in this study according to each organ involvement.

| Characgteristics | Peripheral blood | Bone marrow | Lymph nodes | Skin | Lung | Liver | Spleen | CNS | GI tract | Pleural effusion | Ascites | Kidney | Pancreas | Muscle | Pleura | Peritoneum | Total (n=140) |
| --- | --- | --- | --- | --- | --- | --- | --- | --- | --- | --- | --- | --- | --- | --- | --- | --- | --- |
| Cases with specific organ involvement (/evaluable cases, %) | 107 (/137, 78%) | 68 (/101, 67%) | 121 (/134, 90%) | 68 (/122, 56%) | 32 (/117, 27%) | 59 (/119,50%) | 57 (/119,48%) | 23 (/117, 20%) | 18 (/110, 16%) | 13 (/118, 11%) | 9 (/114, 8%) | 5 (/112, 4%) | 3 (/112, 3%) | 2 (/112, 2%) | 2 (/112, 2%) | 2 (/112, 2%) |  |
| Median age at the start of initial treatment (range) | 63 (28-82) | 63.5 (36-82) | 63 (28-82) | 63 (28-82) | 67 (36-82) | 62 (36-75) | 63 (28-80) | 65 (41-76) | 62 (44-82) | 67 (59-74) | 62 (51-74) | 68 (66-73) | 63 (61-63 | 62.5 (59-66) | 51.5 (44-59) | 66.5 (63-70) | 62 (28-82) |
| Sex, male (%) | 48 (45%) | 32 (47%) | 60 (50%) | 37 (54%) | 16 (50%) | 33 (56%) | 33 (58%) | 14 (61%) | 10 (56%) | 3 (23%) | 1 (11%) | 2 (40%) | 0 (0%) | 2 (100%) | 1 (50%) | 1 (50%) | 68 (49%) |
| ECOG PS; evaluable cases | 99 | 65 | 110 | 60 | 31 | 55 | 54 | 21 | 16 | 12 | 9 | 5 | 3 | 2 | 2 | 2 | 124 |
| 0-1 | 85 (86%) | 57 (88%) | 92 (84%) | 46 (77%) | 23 (74%) | 42 (76%) | 44 (81%) | 14 (67%) | 14 (88%) | 3 (25%) | 5 (56%) | 3 (60%) | 1 (33%) | 1 (50%) | 2 (100%) | 2 (100%) | 100 (81%) |
| ≥2 | 14 (14%) | 8 (12%) | 18 (16%) | 14 (23%) | 8 (26%) | 13 (24%) | 10 (19%) | 7 (33%) | 2 (13%) | 9 (75%) | 4 (44%) | 2 (40%) | 2 (67%) | 1 (50%) | 0 (0%) | 0 (0%) | 24 (19%) |
| Clinical stage, ≤III/IV/NA | 0/107/0 | 0/68/0 | 2/118/1 | 0/68/0 | 0/32/0 | 0/59/0 | 0/57/0 | 0/23/0 | 0/18/0 | 0/13/0 | 0/9/0 | 0/5/0 | 0/3/0 | 0/2/0 | 0/2/0 | 0/2/0 | 2/137/1 |
| simplified ATL-PI; evaluable cases | 97 | 64 | 108 | 59 | 31 | 54 | 53 | 19 | 16 | 12 | 9 | 5 | 3 | 2 | 2 | 2 | 122 |
| Low | 26 (27%) | 13 (20%) | 29 (27%) | 17 (29%) | 4 (13%) | 7 (13%) | 9 (17%) | 4 (21%) | 2 (13%) | 0 (0%) | 1 (11%) | 0 (0%) | 0 (0%) | 1 (50%) | 0 (0%) | 0 (0%) | 33 (27%) |
| Int | 57 (59%) | 43 (67%) | 65 (60%) | 29 (49%) | 16 (52%) | 37 (69%) | 34 (64%) | 8 (42%) | 13 (81%) | 6 (50%) | 4 (44%) | 3 (60%) | 2 (67%) | 1 (50%) | 2 (100%) | 2 (100%) | 70 (57%) |
| High | 14 (14%) | 8 (13%) | 14 (13%) | 13 (22%) | 11 (34%) | 10 (19%) | 10 (19%) | 7 (37%) | 1 (6%) | 6 (50%) | 4 (44%) | 2 (40%) | 1 (33%) | 0 (0%) | 0 (0%) | 0 (0%) | 19 (16%) |
| ATL subtype at the start of initial treatment; evaluable cases | 107 | 68 | 121 | 68 | 32 | 59 | 57 | 23 | 18 | 13 | 9 | 5 | 3 | 2 | 2 | 2 | 140 |
| Chronic (Unfavorable) | 8 (7%) | 0 (0%) | 6 (5%) | 5 (7%) | 2 (6%) | 1 (2%) | 2 (4%) | 0 (0%) | 0 (0%) | 0 (0%) | 0 (0%) | 0 (0%) | 0 (0%) | 1 (50%) | 0 (0%) | 0 (0%) | 8 (6%) |
| Lymphoma | 0 (0%) | 2 (3%) | 13 (11%) | 4 (6%) | 2 (6%) | 3 (5%) | 1 (2%) | 3 (13%) | 4 (22%) | 1 (8%) | 0 (0%) | 1 (20%) | 1 (33%) | 0 (0%) | 0 (0%) | 0 (0%) | 13 (9%) |
| Acute | 99 (93%) | 66 (97%) | 102 (84%) | 59 (87%) | 28 (88%) | 55 (93%) | 54 (95%) | 20 (87%) | 14 (78%) | 12 (92%) | 9 (100%) | 4 (80%) | 2 (67%) | 1 (50%) | 2 (100%) | 2 (100%) | 119 (85%) |
| Initial systemic therapy for aggressive ATL: evaluable cases | 107 | 68 | 121 | 68 | 32 | 59 | 57 | 23 | 18 | 13 | 9 | 5 | 3 | 2 | 2 | 2 | 140 |
| VCAP-AMP-VECP | 73 (68%) | 48 (71%) | 81 (67%) | 42 (62%) | 18 (56%) | 40 (68%) | 38 (67%) | 13 (57%) | 13 (72%) | 6 (46%) | 8 (89%) | 2 (40%) | 2 (67%) | 1 (50%) | 2 (100%) | 1 (50%) | 91 (65%) |
| CHOP like Regimen | 15 (14%) | 11 (16%) | 21 (17%) | 9 (13%) | 6 (19%) | 9 (15%) | 9 (16%) | 2 (9%) | 2 (11%) | 2 (15%) | 0 (0%) | 3 (60%) | 1 (33%) | 1 (50%) | 0 (0%) | 1 (50%) | 22 (16%) |
| Mogamulizumab ± Chemotherapy | 12 (11%) | 8 (12%) | 13 (11%) | 10 (15%) | 5 (16%) | 7 (12%) | 5 (9%) | 4 (17%) | 1 (6%) | 4 (31%) | 1 (11%) | 0 (0%) | 0 (0%) | 0 (0%) | 0 (0%) | 0 (0%) | 17 (12%) |
| Other | 7 (7%) | 1 (1%) | 6 (5%) | 7 (10%) | 3 (9%) | 3 (5%) | 5 (9%) | 4 (17%) | 2 (11%) | 1 (8%) | 0 (0%) | 0 (0%) | 0 (0%) | 0 (0%) | 0 (0%) | 0 (0%) | 10 (7%) |
| Median value of each data |  |  |  |  |  |  |  |  |  |  |  |  |  |  |  |  |  |
| sIL-2R, U/mL | 23,170 | 29,200 | 19,035 | 16,917 | 27,000 | 29,200 | 32,000 | 26,551 | 28,001 | 17,592 | 51,400 | 35,793 | 57,900 | 6695 | 59,250 | 28,997 | 19,035 |
| LDH, IU/L | 519 | 516.5 | 511 | 471 | 551 | 572 | 578 | 600 | 438 | 640 | 965 | 514 | 965 | 222 | 580 | 544 | 508 |
| Serum albumin, g/dL | 3.4 | 3.4 | 3.4 | 3.4 | 2.9 | 3.4 | 3.4 | 3 | 3.2 | 2.75 | 2.5 | 2.6 | 3.1 | 3.5 | 3.3 | 3.1 | 3.4 |
| Corrected Ca level, mg/dL | 9.8 | 10.0 | 9.7 | 9.7 | 10.1 | 9.9 | 9.8 | 10.2 | 9.8 | 10.5 | 9.4 | 12.7 | 10.1 | 9.1 | 10.6 | 10.7 | 9.8 |
| C-reactive protein, mg/dL | 0.47 | 0.43 | 0.64 | 0.52 | 1.56 | 0.90 | 0.64 | 0.66 | 0.47 | 1.33 | 1.08 | 3.96 | 1.49 | 0.83 | 1.55 | 1.84 | 0.59 |
| Cases with allo-HCT (%) | 65 (61%) | 42 (62%) | 71 (59%) | 35 (51%) | 8 (25%) | 32 (54%) | 33 (58%) | 7 (30%) | 9 (50%) | 5 (38%) | 5 (56%) | 2 (40%) | 2 (67%) | 2 (100%) | 0 (0%) | 2 (100%) | 82 (59%) |
| Cases ≤70 years with allo-HCT /evaluable cases (%) | 65/90 (72%) | 42/56 (75%) | 71/103 (69%) | 35/57 (61%) | 8/22 (36%) | 32/52 (62%) | 33/48 (69%) | 7/18 (39%) | 9/14 (64%) | 5/10 (50%) | 5/8 (63%) | 2/4 (50%) | 2/3 (67%) | 2/2 (100%) | 0/2 (0%) | 2/2 (100%) | 82/120 (68%) |
| Median follow-up time, months (range) | 15 (1-206) | 15.5 (1-206) | 16 (0-244) | 13 (0-206) | 7.5 (0-78) | 9 (0-191) | 9 (0-206) | 7 (0-26) | 11 (0-191) | 11 (3-94) | 14 (4-132) | 6 (2-27) | 89 (63-111) | 15.5 (14-17) | 2 (1-3) | 23.5 (20-27) | 15 (0-244) |
| Median overall survival, months (95% confidence interval) | 17.6 (14.7-31.4) | 24.4 (13.3-34.9) | 19.6 (14.5-31.4) | 14.9 (10.0-25.7) | 8.9 (6.1-19.6) | 10.6 (8.7-16.2) | 10.6 (9.0-17.6) | 8.2 (6.7-8.7) | 27.4 (9.6-162.7) | 16.2 (7.6-89.3) | 14.4 (4.4-89.3) | 10.2 (3.5-NA) | 89.3 (63.4-NA) | 16.2 (14.9-NA) | 2.8 (1.8-NA) | 24.1 (20.8-NA) | 20.8 (14.7-31.4) |
| Two-year overall survival, % (95% confidence interval) | 47.9 (37.8-57.3) | 50.5 (37.7-61.9) | 47.1 (37.7-55.9) | 41.3 (29.0-53.1) | 28.1 (12.5-46.1) | 26.2 (15.4-38.3) | 32.2 (20.1-45.0) | 4.9 (0.3-20.0) | 51.4 (35.4-72.4) | 33.2 (8.4-61.2) | 22.2 (3.4-51.3) | 25.0 (0.9-66.5) | NA | NA | NA | 50.0 (0.6-91.0) | 48.2 (39.3-56.5) |
| Cause of Death; all deaths | 68 | 44 | 81 | 45 | 23 | 47 | 41 | 20 | 12 | 8 | 8 | 4 | 3 | 2 | 2 | 2 | 92 |
| ATL | 40 (59%) | 25 (57%) | 54 (67%) | 24 (53%) | 18 (78%) | 29 (62%) | 23 (56%) | 16 (80%) | 6 (50%) | 7 (88%) | 5 (63%) | 3 (75%) | 3 (100%) | 1 (50%) | 2 (100%) | 2 (100%) | 57 (62%) |
| Infection | 12 (18%) | 8 (18%) | 13 (16%) | 10 (22%) | 3 (13%) | 9 (19%) | 9 (22%) | 0 (0%) | 3 (25%) | 1 (13%) | 2 (25%) | 0 (0%) | 0 (0%) | 0 (0%) | 0 (0%) | 0 (0%) | 15 (16%) |
| Organ Failure | 9 (13%) | 5 (11%) | 6 (7%) | 5 (11%) | 1 (4%) | 5 (11%) | 6 (15%) | 3 (15%) | 1 (8%) | 0 (0%) | 1 (13%) | 1 (25%) | 0 (0%) | 1 (50%) | 0 (0%) | 0 (0%) | 10 (11%) |
| Second Cancer | 3 (4%) | 3 (7%) | 3 (4%) | 2 (4%) | 0 (0%) | 1 (2%) | 0 (0%) | 0 (0%) | 1 (8%) | 0 (0%) | 0 (0%) | 0 (0%) | 0 (0%) | 0 (0%) | 0 (0%) | 0 (0%) | 4 (4%) |
| Other | 4 (6%) | 3 (7%) | 5 (6%) | 4 (9%) | 1 (4%) | 3 (6%) | 3 (7%) | 1 (5%) | 1 (8%) | 0 (0%) | 0 (0%) | 0 (0%) | 0 (0%) | 0 (0%) | 0 (0%) | 0 (0%) | 6 (7%) |

allo-HCT, allogeneic hematopoietic cell transplantation;ATL, adult T-cell leukemia/lymphoma; Ca, calcium; CHOP, cyclophosphamide, vincristine, doxorubicin, and prednisolone; CNS, central nervous system; ECOG PS; Eastern Cooperative Oncology Group performance status; GI tract, gastrointestinal tract; LDH, lactate dehydrogenase; NA, not available; PI, prognostic index; sIL-2R, soluble interleukin-2 receptor; VCAP, vincristine, cyclophosphamide, doxorubicin, and prednisolone, -AMP, doxorubicin, ranimustine, and prednisolone, -VECP, vindesine, etoposide, carboplatin, and prednisolone.

Table S4. Univariate and multivariate analysis: effects of organ involvements on overall survival.

| Organ involvement  positive vs negative | Univariate analysis | | Multivariate analysis | |
| --- | --- | --- | --- | --- |
|  | **Hazard ratio (95% CI)** | ***P* value** | **Hazard ratio (95% CI)** | ***P* value** |
| Lung | 2.44 (1.41-4.22) | 0.002 | 2.15 (1.18-3.91) | 0.012 |
| Liver | 2.66 (1.60-4.44) | <0.001 | 2.13 (1.23-3.68) | 0.007 |
| Spleen | 2.06 (1.24-3.41) | 0.005 | 0.98 (0.53-1.81) | 0.953 |
| Central nervous system | 6.11 (3.28-11.38) | <0.001 | 4.87 (2.43-9.79) | <0.001 |

CI, confidence interval

Table S5. Univariate and multivariate analysis: effects of each parameter on overall survival.

|  | Univariate analysis | | Multivariate analysis | |
| --- | --- | --- | --- | --- |
|  | **Hazard ratio (95% CI)** | ***P* value** | **Hazard ratio (95% CI)** | ***P* value** |
| Age (>70 vs ≤70, years) | 1.69 (0.87-3.26) | 0.122 | 1.73 (0.82-3.67) | 0.513 |
| ECOG PS (≥2 vs 0-1) | 1.99 (1.09-3.63) | <0.001 | 1.74 (0.86-3.54) | 0.126 |
| ATL subtype (acute vs other) | 0.82 (0.42-1.59) | 0.556 | 0.76 (0.37-1.56) | 0.452 |
| Serum albumin level (≥3.5 vs <3.5, g/dL) | 2.83 (1.69-4.81) | <0.001 | 1.71 (0.86-3.40) | 0.125 |
| sIL-2R level (>20,000 vs ≤20,000, U/mL) | 1.74 (1.05-2.88) | 0.033 | 1.19 (0.67-2.10) | 0.552 |
| Corrected serum Ca level (≥11 vs <11, mg/dl) | 1.40 (0.66-2.95) | 0.383 | 1.05 (0.47-2.34) | 0.908 |
| C-reactive protein level (≥2.5 vs <2.5, mg/dL) | 1.82 (1.01-3.27) | 0.048 | 1.38 (0.65-2.92) | 0.399 |
| Risk organ involvement (positive vs negative) | 3.07 (1.74-5.44) | <0.001 | 2.50 (1.34-4.70) | 0.004 |

ATL, adult T-cell leukemia/lymphoma; Ca, calcium; CI, confidence interval; ECOG PS; Eastern Cooperative Oncology Group performance status; sIL-2R, soluble interleukin-2 receptor
