# Supplementary material for: Prognostic impact of organ involvement in aggressive adult T-cell leukemia/lymphoma: definition of risk organ and proposal of a prognostic index
Source: Blood Cancer J. 2025 Oct 16;15(1):166. doi: 10.1038/s41408-025-01367-w (PMC12533117; doi:10.1038/s41408-025-01367-w)
Supplement: Supplementary file 3 — Supplemental methods and discussion [file 41408_2025_1367_MOESM3_ESM.docx]

**Supplemental Materials**

**Methods**

**Patients, clinical data, and definitions**

Aggressive adult T-cell leukemia/lymphoma (ATL) cases (acute, lymphoma, and chronic types with unfavorable factors) treated at the Research Hospital, Institute of Medical Science, University of Tokyo, between April 2004 and November 2024 were included. The clinical subtypes of ATL were classified based on the Shimoyama classification.^1^ Clinical stage was classified using the Ann Arbor staging classification.^2^ Definitions of each organ involvement are shown in Table S2. Cases in which the presence or absence of specific organ lesions could not be determined were excluded from the related analyses. All clinical information was extracted from medical records. The institutional review board of the Institute of Medical Science, University of Tokyo, approved this study (2024-74-1211).

**Statistical analysis**

The log-rank test was used for univariate analysis of overall survival (OS). The Cox proportional hazards model was used for univariate and multivariate analyses of OS and reported hazard ratios and concordance indices (C-indices). A multivariate analysis was performed to estimate risk organ involvement, including variables for organ involvement with an incidence greater than 5% and significant impact in the univariate analysis. For the multivariate analysis estimating factors affecting OS in this cohort, we included, in addition to the presence or absence of risk organs, components of previously reported prognostic models^3-5^ that were applicable to at least 5% of cases. Cases with missing data for a particular analysis were excluded from that analysis. All statistical analyses were performed using GraphPad Prism software (version 7.0d; GraphPad Software), or EZR (version 1.55; Saitama Medical Center, Jichi Medical University).^6^ All *P* values were two-sided, and *P* values < 0.05 were considered statistically significant.

**Supplemental discussion**

In this study, we clarified the association between organ involvement profiles and prognosis based on detailed information from aggressive ATL patients. We identified risk organs whose involvement is associated with poor prognosis and proposed a novel prognostic model, the risk organ index for aggressive ATL.

Several cohorts have shown some organ involvement profiles of patients with aggressive ATL,^3, 4, 7, 8^ and it is recognized that aggressive ATL often presents with a variety of organ lesions. However, there has been no detailed and comprehensive assessment system of systemic organ involvement defined by uniform criteria as presented in this report. It is noteworthy that the percentage of each organ involvement in this report is relatively high compared to previous studies. There are several possible reasons for this. First, as is often the case in multicenter reports, the criteria for each organ involvement are not clearly defined. Second, some study cohorts excluded certain cases due to inclusion and exclusion criteria, which may have affected the reported rates of organ involvement. Most significantly, this report excluded cases where the presence or absence of relevant organ lesions could not be determined, whereas previous reports likely did not apply this exclusion.^3, 4, 8^ Considering these factors, this report provides useful data showing the detailed rates of systemic organ involvement in aggressive ATL.

Although the details of organ involvement profiles and their prognostic impact had not been clarified in aggressive ATL, this report shows that the prognosis is poor in cases with involvement of multiple organs, particularly those with involvement of specific risk organs (lung, liver, and central nervous system [CNS]). Previous reports showed that aggressive ATL cases with advanced clinical stage, high soluble interleukin-2 receptor levels, and high ATL cell counts in peripheral blood have a poor prognosis.^3, 5, 9^ These findings suggest that systemic tumor burden and the spread of ATL lesions are prognostic factors. Our results support these previous findings by showing that patients with more involved organs have a poorer prognosis. Additionally, it is known that in other lymphomas such as diffuse large B-cell lymphoma and peripheral T-cell lymphoma, cases with more than a certain number of involved organs have a worse prognosis.^10-12^ Thus, it is not surprising that the same is true in aggressive ATL. However, it is important to note that in aggressive ATL, which is mostly classified as stage IV, the prognostic relevance of organ involvement that subdivides stage IV may be more informative than in other lymphomas.

Although the matrices appear similar, involvement of specific risk organs provides a different perspective from simply having a large tumor burden in terms of prognosis. Specifically, involvement of the CNS, liver, and lung is associated with higher prognostic impact than involvement of other organs in ATL cases, and several factors may explain this. First, the blood-brain barrier limits the ability of chemotherapeutic agents to reach the CNS.^13^ Although different treatment approaches are applied for CNS involvement to overcome this barrier, treatment outcomes remain unsatisfactory. Second, patients with liver involvement may have liver dysfunction, which can restrict chemotherapy options or lead to poor drug metabolism, resulting in severe adverse reactions. Third, patients with lung involvement may develop respiratory failure, which may complicate the selection of drugs, including chemotherapy agents, that are toxic to the lungs and circulatory system. Finally, all these risk organs are vital organs, and dysfunction caused by tumor involvement may directly impact prognosis. The high incidence of ATL-associated deaths in cases with CNS and lung involvement (Figure S2C) supports these assumptions. Additionally, lung and CNS involvement were associated with poor Eastern Cooperative Oncology Group performance status (ECOG PS), which may explain the lower proportion of patients with these lesions receiving allo-HCT (Figure S2A, D). Besides, tumor cell involvement of the CNS, lungs, and liver has also been reported to be associated with poor prognosis in other hematological malignancies,^14, 15^ and subsequent dysfunction of these organs may be related to poor prognosis in hematological malignancies. In contrast to these *bona fide* risk organs, spleen involvement was not an independent predictor of shorter survival in the multivariate analysis. This is likely because spleen involvement was strongly associated with involvement of other risk organs (Figure S3) and may have indirectly represented their risk. Additionally, the spleen is not an indispensable organ, and its dysfunction is not typically fatal.

**References**

1. Shimoyama M. Diagnostic criteria and classification of clinical subtypes of adult T-cell leukaemia-lymphoma. A report from the Lymphoma Study Group (1984-87). *British Journal of Haematology* 1991 Nov; **79**(3)**:** 428-437.

2. Carbone PP, Kaplan HS, Musshoff K, Smithers DW, Tubiana M. Report of the Committee on Hodgkin's Disease Staging Classification. *Cancer Res* 1971 Nov; **31**(11)**:** 1860-1861.

3. Katsuya H, Yamanaka T, Ishitsuka K, Utsunomiya A, Sasaki H, Hanada S*, et al.* Prognostic Index for Acute- and Lymphoma-Type Adult T-Cell Leukemia/Lymphoma. *Journal of Clinical Oncology* 2012 May; **30**(14)**:** 1635-1640.

4. Fukushima T, Nomura S, Shimoyama M, Shibata T, Imaizumi Y, Moriuchi Y*, et al.* Japan Clinical Oncology Group (JCOG) prognostic index and characterization of long-term survivors of aggressive adult T-cell leukaemia-lymphoma (JCOG0902A). *British Journal of Haematology* 2014 Sep; **166**(5)**:** 739-748.

5. Fuji S, Yamaguchi T, Inoue Y, Utsunomiya A, Moriuchi Y, Uchimaru K*, et al.* Development of a modified prognostic index for patients with aggressive adult T-cell leukemia-lymphoma aged 70 years or younger: possible risk-adapted management strategies including allogeneic transplantation. *Haematologica* 2017 Jul; **102**(7)**:** 1258-1265.

6. Kanda Y. Investigation of the freely available easy-to-use software 'EZR' for medical statistics. *Bone Marrow Transplant* 2013 Mar; **48**(3)**:** 452-458.

7. Oliveira PD, Farre L, Bittencourt AL. Adult T-cell leukemia/lymphoma. *Rev Assoc Med Bras (1992)* 2016 Oct; **62**(7)**:** 691-700.

8. Katsuya H, Ishitsuka K, Utsunomiya A, Hanada S, Eto T, Moriuchi Y*, et al.* Treatment and survival among 1594 patients with ATL. *Blood* 2015 Dec 10; **126**(24)**:** 2570-2577.

9. Jimbo K, Kawamata T, Inamoto Y, Ito A, Yokoyama K, Sato A*, et al.* Flow cytometric profiles with CD7 and CADM1 in CD4+ T cells are promising indicators for prognosis of aggressive ATL. *Blood Advances* 2024 2024-07-15; **8:** 3760-3770.

10. Project IN-HsLPF. A predictive model for aggressive non-Hodgkin's lymphoma. *N Engl J Med* 1993 Sep 30; **329**(14)**:** 987-994.

11. Gallamini A, Stelitano C, Calvi R, Bellei M, Mattei D, Vitolo U*, et al.* Peripheral T-cell lymphoma unspecified (PTCL-U): a new prognostic model from a retrospective multicentric clinical study. *Blood* 2004 Apr 01; **103**(7)**:** 2474-2479.

12. El-Galaly TC, Villa D, Alzahrani M, Hansen JW, Sehn LH, Wilson D*, et al.* Outcome prediction by extranodal involvement, IPI, R-IPI, and NCCN-IPI in the PET/CT and rituximab era: A Danish-Canadian study of 443 patients with diffuse-large B-cell lymphoma. *Am J Hematol* 2015 Nov; **90**(11)**:** 1041-1046.

13. Arvanitis CD, Ferraro GB, Jain RK. The blood-brain barrier and blood-tumour barrier in brain tumours and metastases. *Nat Rev Cancer* 2020 Jan; **20**(1)**:** 26-41.

14. Zhou Z, Sehn LH, Rademaker AW, Gordon LI, Lacasce AS, Crosby-Thompson A*, et al.* An enhanced International Prognostic Index (NCCN-IPI) for patients with diffuse large B-cell lymphoma treated in the rituximab era. *Blood* 2014 Feb 06; **123**(6)**:** 837-842.

15. Weitzman S, Egeler RM. Langerhans cell histiocytosis: update for the pediatrician. *Curr Opin Pediatr* 2008 Feb; **20**(1)**:** 23-29.
